# Broadband modulation of terahertz waves through electrically driven hybrid bowtie antenna-VO_2_ devices

**DOI:** 10.1038/s41598-017-13085-w

**Published:** 2017-10-05

**Authors:** Chunrui Han, Edward P. J. Parrott, Georges Humbert, Aurelian Crunteanu, Emma Pickwell-MacPherson

**Affiliations:** 1 Department of Electronic Engineering, The Chinese University of Hong Kong Shatin, New Territories, Hong Kong China; 2 XLIM Research Institute, University of Limoges, Limoges, France

## Abstract

Broadband modulation of terahertz (THz) light is experimentally realized through the electrically driven metal-insulator phase transition of vanadium dioxide (VO_2_) in hybrid metal antenna-VO_2_ devices. The devices consist of VO_2_ active layers and bowtie antenna arrays, such that the electrically driven phase transition can be realized by applying an external voltage between adjacent metal wires extended to a large area array. The modulation depth of the terahertz light can be initially enhanced by the metal wires on top of VO_2_ and then improved through the addition of specific bowties in between the wires. As a result, a terahertz wave with a large beam size (~10 mm) can be modulated within the measurable spectral range (0.3–2.5 THz) with a frequency independent modulation depth as high as 0.9, and the minimum amplitude transmission down to 0.06. Moreover, the electrical switch on/off phase transition depends very much on the size of the VO_2_ area, indicating that smaller VO_2_ regions lead to higher modulation speeds and lower phase transition voltages. With the capabilities in actively tuning the beam size, modulation depth, modulation bandwidth as well as the modulation speed of THz waves, our study paves the way in implementing multifunctional components for terahertz applications.

## Introduction

The terahertz (THz) frequency regime attracts attention from both the scientific research and industry sectors due to its technological potential in biomedicine^1,2^, security^3^, imaging^4^ and material characterizations^5^. The terahertz components, such as modulators^6,7^, switches^8,9^, lenses^10^, waveplates^11^, and filters are essential in efficiently manipulating the THz waves for specific applications. The fast growth of the THz technology asks for not only construction but also daily improvement of the functional components.

One significant terahertz device, the optoelectronic compatible THz modulator, plays a key role in THz imaging and wireless communication systems. The establishment of active THz modulators require active materials working at THz frequencies. It is well known that the semiconducting heterostructures are THz active due to their intersubband transitions taking place on a meV energy scale^12,13^. The unique scattering behaviors, such as strong absorption and reflection, induced by the coupling of THz pulses to the intersubband transitions enable implementation of an active THz attenuator, filter or emitter. Additionally, the charge density on the semiconductor surface can be tuned through either electrostatic gating^14^ or photo-excited interband transitions^15^, leading to real time control of THz waves. Similarly, graphene has been recognized as a THz active material since graphene electrons have strong intraband transitions at terahertz frequencies^16,17^. The transition rate can be changed by shifting the Fermi energy of graphene optically or electrically such that the absorption/reflection of THz waves during/after the transition can be modulated in an active way^18–20^. Both semiconductors and graphene are desirable in controlling the charge density and hence the electrical conductivity of the active material, but the tunable range is restricted by either the ultrathin two dimensional electron gas interface or the atomic thin graphene single layer, hence the experimental modulation depth is limited to ~0.5. Metamaterials with scalable geometry are excellent in amplitude, frequency and polarization manipulation of electromagnetic waves^21–23^ but lose the dynamic real-time tunability. Lots of efforts are underway through effective combination of metamaterials with semiconductors^24–26^ and graphene^27,28^. The achievements are obvious but most of them suffer from narrow bandwidth because the electric transition and the subsequent coupling with the metamaterial resonance are always frequency dependent. To achieve ulrabroadband THz modulation without compromise of the modulation depth, speed, beam size as well as real time control, new active materials as well as precise metamaterial designs are required.

The close to room temperature metal-insulator phase transition (MIT) in vanadium dioxide (VO_2_) opens a new path towards multifunctional THz modulators. Firstly, the optical phonon resonance of VO_2_ is far away from the terahertz frequency regime, thereby allowing an ultrabroadband response to be achieved across the whole THz spectral range^29,30^. Secondly, the extreme change of the dielectric property from insulator to metal during structural transition from monoclinic to rutile leads to sharp change in the electrical conductivity of up to 5 orders of magnitude, and hence allows one of the best modulation depth among most of the THz active materials^31^. Thirdly, the precise thickness control along with the guaranteed quality of VO_2_ films benefiting from the advanced growth techniques allow not only tunable modulation depths but also desirable modulation speeds. Fourthly, among the different ways in triggering the MIT transition, electrical switching is highly compatible and stable compared to the optical excitation, which also occurs on faster time scales than the thermal activation^32^. As a result, electric field driven phase transition in VO_2_ provides a good basis for implementing multiple functional THz modulators.

In this article, we demonstrate low voltage, sharp switching broadband terahertz modulator devices based on bowtie-antenna arrays coupled with vanadium dioxide films/wires, and fabricated on c-cut sapphire substrate. Highly efficient modulation can be achieved through all of the measured spectrum from 0.3 to 2.5 THz even though the operability can be extended to much higher frequencies. The modulation depth which is ~0.8 for 170 nm bare VO_2_ film, can be firstly improved to 0.85 by patterning the parallel antenna wires (180 nm thick) on top of VO_2_ and then enhanced to 0.9 by inserting specific bowties in between the antenna wires. The anisotropic configuration of the antenna wire array allows the polarization manipulations of THz waves for which the polarization perpendicular to the wire will be modulated sharply during MIT transition while the one parallel with the wire would be always highly reflected. Furthermore, it is observed that the electrically driven phase transition depends very much on the size of the VO_2_ material, indicating that smaller VO_2_ patterns leads to higher modulation speed and lower phase transition voltage. In short, by combining the advantages of VO_2_ and bowtie antenna array, multiple functions, i.e. ultra-broad bandwidth, high modulation depth, highly tunable modulation speed as well as beam size can be collected in a single electrically driven bowtie antenna-VO_2_ hybrid device.

## Design and Methods

**Figure 1:**
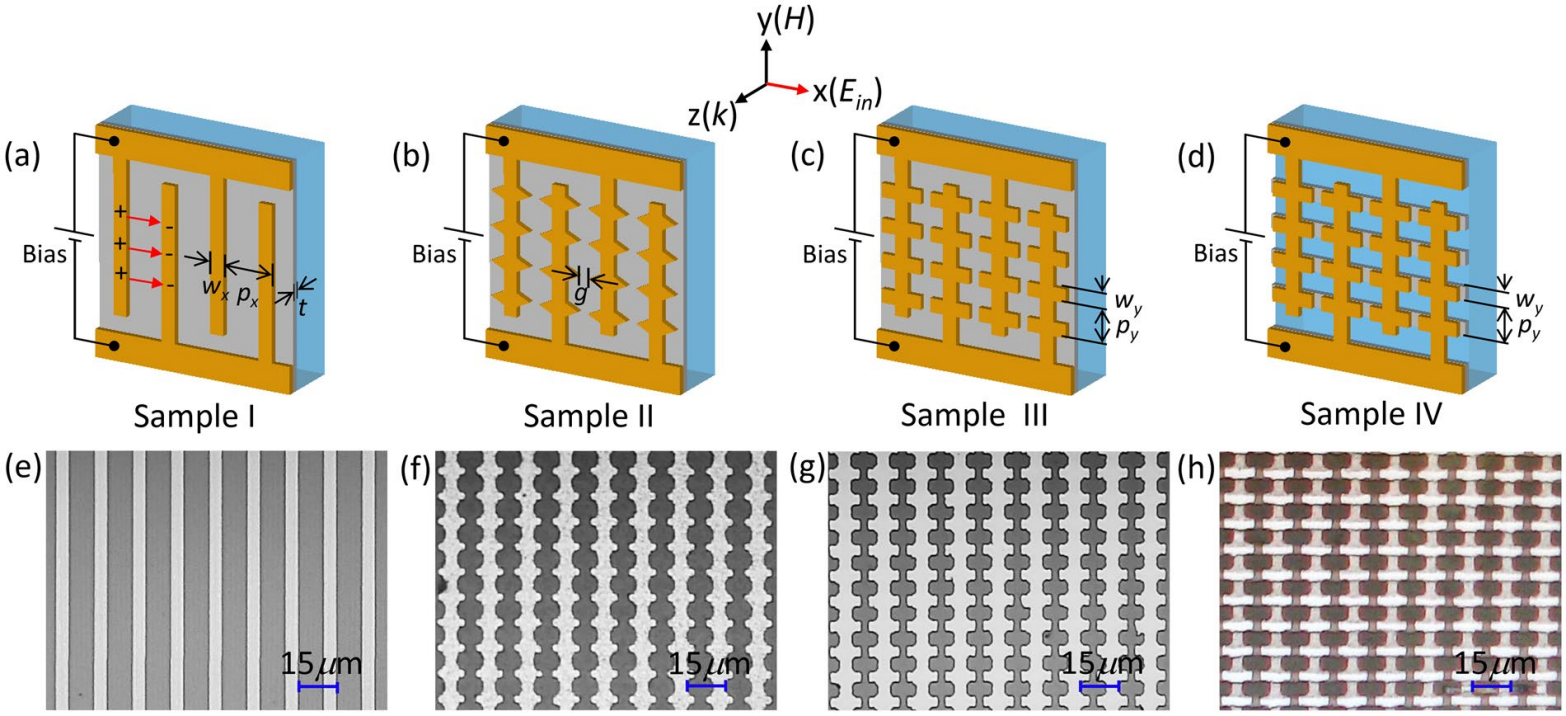
Design and fabrication of the electrically driven hybrid bowtie antenna-VO_2_ devices. (**a**–**d**) Sketches of the four types of sample investigated: Sample I, Al wires on VO_2_ film; Sample II, Al wires with triangle bowtie on VO_2_ film; Sample III, Al wires with square bowtie on VO_2_ film; Sample IV, Al wires with square bowtie on VO_2_ wires. Color code: orange for aluminum, gray for VO_2_ and blue for c-sapphire substrate. (**e**–**h**) The corresponding optical microscope images. The geometry parameters for the four types of sample are summarized in Table 1. The incident electric field *E*_*in*_ is linearly polarized in the *x* direction. The electrical activation was realized by applying an external voltage bias between adjacent Al wires. Red arrows in (**a**) indicate the electric field built through the charge accumulations on two neighbouring wires.

The VO_2_ layers have been fabricated in advance on c-cut sapphire substrate at 500 °C, using reactive electron-beam evaporation of a vanadium target in an oxygen atmosphere^33^. The metallic antenna with different bowties, wire antenna (First column of Fig. 1), wire antenna with triangle bowties (Second column of Fig. 1), wire antenna with square bowties (Third and fourth columns of Fig. 1) were defined by optical lithography using electron-beam evaporation of aluminum and the lift-off method. The VO_2_ wires in Fig. 1(h) were fabricated from a planar VO_2_ layer which was patterned using a lithographically-defined photo-resist mask and a wet etching process. The geometry parameters of the four types fabricated samples are summarized in Table 1.

**Table 1:** The geometry parameters of the four types of sample as shown in Fig. 1. In all cases the samples were prepared on c-sapphire substrates and the metal thickness was 180 nm.

Sample Type	*P*_*x*_ (*μ*m)	*P*_*y*_ (*μ*m)	*w*_*x*_ (*μ*m)	*w*_*y*_ (*μ*m)	*g* (*μ*m)	*t* (nm)
I	15		5			170
II	15	10	5	4	5	170
III	15	10	5	4	3	170
IV	15	10	4	4	4	200

Terahertz spectroscopy was performed using the apparatus and methods described previously^34^. For the electrical field based measurements, an external voltage was applied to the well-defined Al electrodes at the extremities of the antenna array (Fig. 1(a)). The positive and negative charges would then accumulate on the adjacent antenna lines to build an electric field perpendicular to the wires (red arrows in Fig. 1(a)). By increasing the external voltage, the electric field between the wires increases as well, leading to break down of VO_2_ insulating state and subsequently large current jumps in the I(V) curves, indicative of the VO_2_ transition to the metallic state.

A commercial-grade simulator based on the finite-difference time-domain method was used to calculate the time-domain response of a broadband terahertz pulse propagating through the bowtie-antenna VO_2_ hybrid structures^35^. From these time-domain results the frequency response of the structures were derived as for the experimental results by Fourier transforming the propagating result and comparing this with the input.

## Results and Discussions

### Bowtie arrays on top of VO_2_ film

The bowtie arrays consisting of metallic wires in the vertical direction and bowties in between the metallic wires in the horizontal direction play four important roles. Firstly, the antenna wires serve as electrodes for building up the electric field to break down the VO_2_ film and induce the MIT transition; hence they are indispensable in the hybrid metal-VO_2_ device. Secondly, they are able to enhance the modulation depth of bare VO_2_ film. The effect has been demonstrated in thermally activated metal-VO_2_ wire grid structures^36,37^ which exhibit different degrees of enhancement depending on the conductivity of the VO_2_ and the thickness of the metallic wire grid. Thirdly, the orientation of metallic array is designed to fit the dipolar characteristics of the THz pulse, for which only the TM polarization mode, defined as the incident electric field perpendicular to the Al wires, is allowed to be highly modulated. Fourthly, the modulation area defined by the size of the array can be changed from micrometers to a few centimeters.

**Figure 2:**
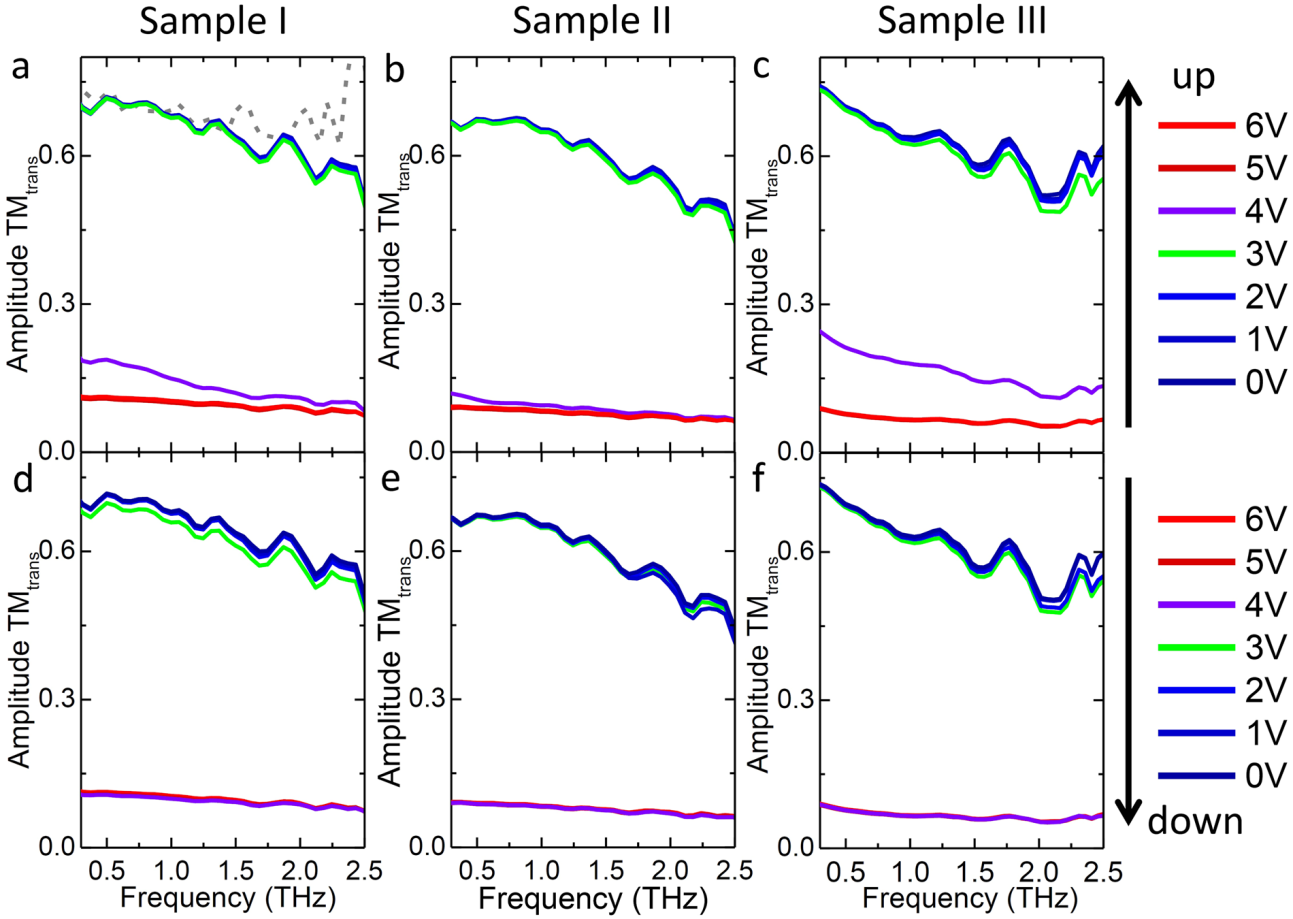
Measured amplitude transmission for the TM mode for the sample I-III. The top row **(a**–**c)** is when the applied voltage was increased; the bottom row **(d**–**f)** is when the applied voltage was decreased. The grey dashed curve in **(a)** indicates the amplitude transmission of 170 nm bare VO_2_ measured at 20 °C.

Two processes, one for voltage going up and the other for the voltage going down, were measured with the corresponding TM transmission for the three bowtie metallic arrays shown in the top and bottom row of Fig. 2, respectively. The THz beam path was purged with dry nitrogen in order to avoid absorption by water vapor. It is interesting to note that the amplitude transmission of the three devices are remarkably similar. All of them exhibit ultrabroadband responses with similar trends for the amplitude transmission curves and display similar switching on voltages around 4 V, where the amplitude transmission drops sharply as shown by the purple curves in Fig. 2(a–c), indicating that insulator to metal transition (IMT) is triggered at this voltage. Additionally, the threshold voltage for the metal to insulator transition (MIT) occurs at 3 V where the amplitude transmission returns to the higher value without any intermediate transmission levels as shown by the green curves in Fig. 2(d–f). The relatively similar behaviors for the amplitude transmission are determined by the same thickness of the VO_2_ film and the same, or similar, dimension/periods of the metallic arrays. It is notable that the insertion loss due to the metallic coating on top of the VO_2_ is small since the amplitude transmission of the devices in the insulating state is close to that of the bare VO_2_ at the room temperature (grey dashed curve in Fig. 2(a)). This results from the specific orientation of the antenna array, which is chosen to fully allow the transmission of the TM polarization mode with an incident electric field perpendicular to the metallic wires.

**Figure 3:**
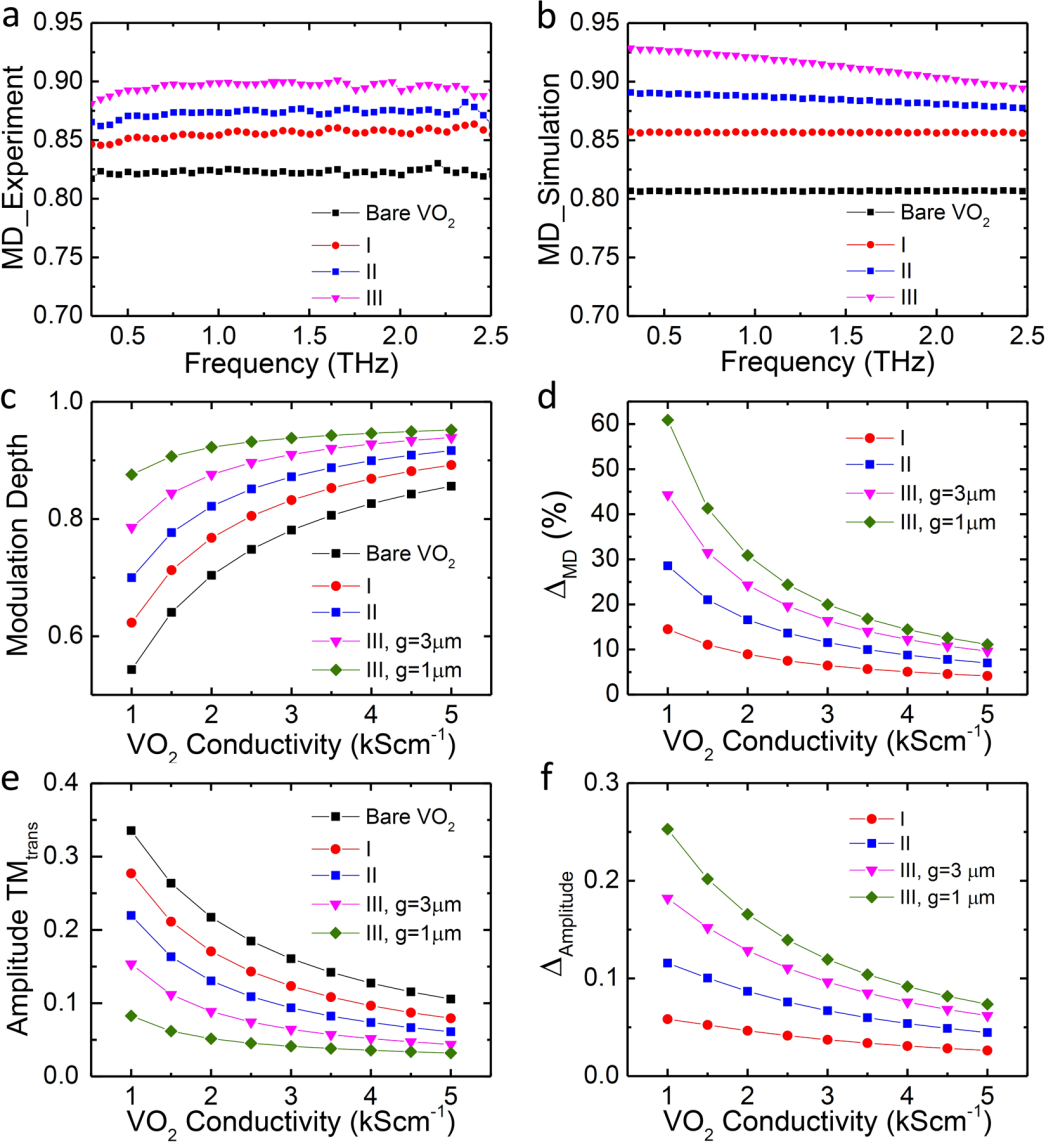
Modulation depth characterization. (**a**) The experimental modulation depth for bare VO_2_ by thermal activation (black squares), sample I (red dots), sample II (blue squares) and sample III (pink triangles) by electrical activation. (**b**) The corresponding simulated MD. (**c**) Simulated modulation depth at 1 THz for bare VO_2_ (black squares), sample I (red dots), sample II (blue squares), sample III with g = 3 μm (pink triangles) and 1 μm (green diamonds), respectively, as a function of VO_2_ conductivity. (**d**) Increase in MD for different types of sample compared to bare VO_2_. (**e**) Simulated TM amplitude transmission at 1 THz as a function of VO_2_ conductivity. (**f**) Decrease in amplitude transmission for different types of sample compared to bare VO_2_ at 1 THz as a function of VO_2_ conductivity.

To characterize the efficiency of the hybrid bowtie metallic-VO_2_ terahertz modulators, the modulation depth (MD), defined as MD = (*E*_*i*_ − *E*_*m*_)/*E*_*i*_, is calculated as a function of operation frequency, where *E*_*i*_ and *E*_*m*_ are the transmission amplitude at the insulator and metal state, respectively. We observe very good broadband responses from 0.3 to 2.5 THz that are presented clearly by the flat experimental MDs as shown in Fig. 3(a). They are fitted well by the numerical simulations in Fig. 3(b) when the conductivity of VO_2_ film at metallic state is set to be σ = 3.5 kScm^−1^. Moreover, the experimental MD changes for different sample types depending on the metallic structure. Specifically, it is ~0.82 for 170 nm thick bare VO_2_ film (black square curve in Fig. 3(a)) through thermal activation. This value increases to 0.85 (red dots), 0.87 (blue squares) and 0.9 (pink triangles) step by step when the 180 nm thick Al wires, triangle bowties with Al wires and square bowties with Al wires are patterned on top of the VO_2_ film, respectively, manifesting the enhancement effect of each component in the bowtie array. Due to the design of the metamaterials, the enhancement in MD from the three metamaterial structures is a result of decreased transmission in the metallic state resulting from the metamaterial structures; *E*_*i*_ for all three metamaterial structures are similar to the transmission for bare VO_2_ as seen in Fig. 2(a–c).

To have a complete characterization for this enhancement effect, we calculated the MD of different types of sample as a function of conductivity as shown in Fig. 3(c). With increasing conductivity, the MDs increase for all of the sample types, but the rising trends are quite different. The bare VO_2_ film with smallest MD has the largest increase from 0.54 to 0.85 (black squares in Fig. 3(c)) as the conductivity increases from 1 to 5 kScm^−1^, while sample III with a 1 *μ*m gap exhibits the highest MD but the slowest rise from 0.88 to 0.95; indicating that the enhancement effect due to patterning metallic structures on top of VO_2_ is much more dramatic at low conductivity than at high conductivity. To quantify such an enhancement, the increases in MD, defined as *Δ*_MD_ = (MD_metal on VO2_ − MD_bare VO2_)/MD_bare VO2_, for samples I-III compared to the bare VO_2_, are plotted in Fig. 3(d). The *Δ*_MD_ increases step by step from sample I to sample III, which is 5.6%, 10% and 14%, respectively, as shown by the red dot, blue square and pink triangle at σ = 3.5 kScm^−1^ in Fig. 3(d). These values are slightly larger than the experimental results (3.8%, 6.1% and 9.2%) mainly due to the structure tolerance of bowtie-metallic arrays during the fabrication. Different gap sizes between square bowties of sample III with 3 and 1 *μ*m respectively are also compared as shown by the pink triangles and green diamonds of Fig. 3(d), indicating that a smaller gap leads to higher *Δ*_MD._ The *Δ*_MD_ increases as the VO_2_ conductivity decreases which is consistent with the former calculations for the wire-grid structure on top of VO_2_ by thermal activation^36^. Importantly, this MD improvement increases from sample I to sample III with the largest *Δ*_MD_ ~60% achieved in the square bowtie-metallic array with a 1 *μ*m gap and a 1 kScm^−1^ VO_2_ conductivity (green diamonds in Fig. 3(d)). In short, among the different metallic array designs studied herein, the square bowtie metallic array (sample III) on top of VO_2_ exhibits highest modulation depth and least dependence on the VO_2_ conductivity (Fig. 3(c)).

To find the origin of the enhancement behavior, the amplitude transmission of different types of sample at 1 THz as a function of VO_2_ conductivity is plotted in Fig. 3(e). The amplitude transmission decreases gradually from sample I to sample III compared to bare VO_2_, which is 0.107, 0.081, 0.058 at σ = 3.5 kScm^−1^ respectively as shown by the red dot, blue square and pink triangle in Fig. 3(e). These values have a good agreement with the experimental results (0.095, 0.081 and 0.064) for the amplitude transmission of sample I-III in Fig. 2. Such decreases in the amplitude transmission cause the step by step enhancement of the MD in the different metallic arrays as shown in Fig. 3(c). The sample III with 1 *μ*m gap has smallest amplitude transmission (green diamonds in Fig. 3(e)) which leads to the highest modulation depth in Fig. 3(c). As the conductivity of VO_2_ increases, the amplitude transmission of the square bowtie metallic arrays (pink triangles and green diamonds in Fig. 3(e)) decrease less rapidly than the other samples, resulting in the MD having a lower dependence on the VO_2_ conductivity as shown in Fig. 3(c). This lower dependence would allow a thinner VO_2_ film to be utilized, as film conductivity depends somewhat on VO_2_ film thickness^38^. The decrease for the amplitude transmission relative to bare VO_2_, defined as *Δ*_Amplitude_ = *E*_VO2_ − *E*_metal on VO2_, is calculated and shown in Fig. 3(f). Sample III with a 1 *μ*m gap (green diamonds in Fig. 3(f)) exhibits the largest *Δ*_Amplitude_, resulting in the highest *Δ*_MD_ in Fig. 3(d), while sample I with the largest gap of 10 *μ*m produces the smallest *Δ*_Amplitude_ (red dots in Fig. 3(c)) and hence the lowest *Δ*_MD_ in Fig. 3(d). These behaviors indicate that the amplitude transmission depends very much on the gap size between the bowties, with a smaller amplitude transmission produced by a smaller gap. This is because when the VO_2_ is switched to the metallic phase, the bowtie antennae mimic a wire grid orthogonal to the metal wire grids of the design. Consequently, the device acts as a pair of crossed polarizers, reducing transmission.

**Figure 4:**
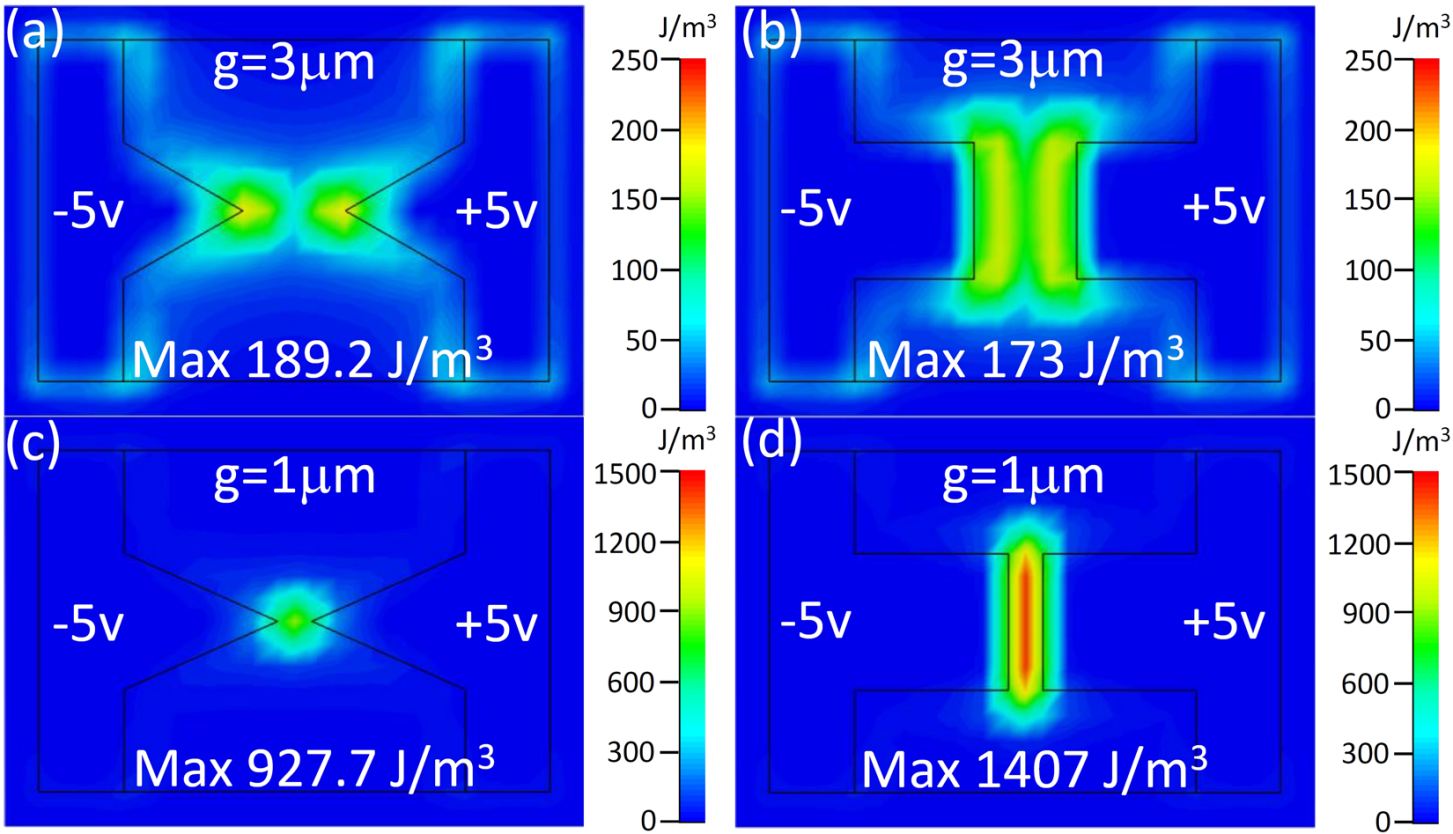
Simulated distributions of the electric energy density at the interface of metallic array and VO_2_ film. ±5 V voltages are applied on the left and right hand side electrodes, respectively, for 3 μm gap (**a**) triangle and (**b**) square bowties, and 1 μm gap (**c**) triangle and (**d**) square bowties.

To understand the effects of gap size and bowtie shape on the VO_2_ transition, the distributions of the electric energy density at the interface of the metallic array and the VO_2_ film are shown in Fig. 4. For the triangle bowtie antenna, the maximum electric energy density increases from 189.2 to 927.7 J/m^3^ when the gap size decreases from 3 to 1 *μ*m whereas the square bowtie antenna energy density increases from 173 to 1407 J/m^3^. Hence, the maximum electric energy density increases by five and eight times for triangle and square shaped antennae respectively, indicating that the square bowtie with the smaller gap develops the highest electrical field concentration. Additionally, it is clear that the square bowties shown in Fig. 4(b) and (d) have a larger effective field area applied to the VO_2_ film than the triangle bowties shown in Fig. 4(a) and (c). As a result, the square bowtie with smaller gap size is the most promising configuration to achieve both high modulation depth and fast switching of the VO_2_ phase transition.

### Square bowtie-metallic arrays on top of VO_2_ wires

#### Transmission amplitude and modulation depth

**Figure 5:**
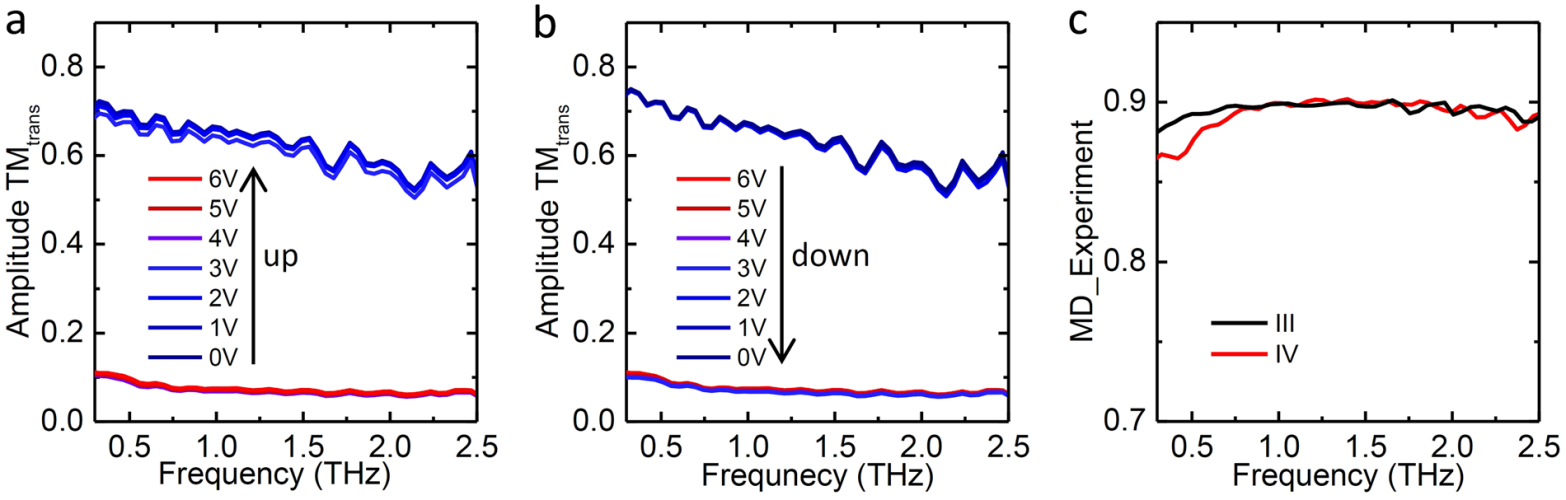
THz measurement for sample IV. Measured amplitude transmission for the TM mode with voltage going up (**a**) and down (**b**), respectively. (**c**) Experimental modulation depth of sample III (black curve) and sample IV (red curve).

To investigate the configuration effect of VO2 on the modulation performance of the hybrid metal-VO_2_ device, the VO_2_ film of sample III was replaced by parallel wires directly underneath the square bowties to form sample IV (Fig. 1(d) and (h)). The amplitude transmission of the TM mode for voltage ramp up and down is shown in Fig. 5(a) and (b) respectively. The sharp decrease and increase in the amplitude transmission at the switching voltages (4 V and 2 V for switching on and off, respectively) are similar to the behaviors observed in samples I-III (Fig. 2), but different from the behaviors of bare VO_2_ film and wire grid-VO_2_ devices^36^ by thermal activations in which several levels of transmission amplitude can be recorded within the temperature range of the first-order metal-insulator transition. This difference displays a key advantage of the electrically driven phase transition of VO_2_ in that it is much faster than thermal activation. When the threshold voltage for the MIT in VO_2_ is achieved, the current within the overall device increases/decreases dramatically and abruptly, leading to the phase transition within a few seconds, which is beyond the temporal resolution of our THz measurement setup. It is notable that the MD of sample IV (red curve in Fig. 5(c)) is as high as that of sample III (black curve in Fig. 5(c)) even though the VO_2_ wires now cover much less of the sample area than the earlier VO_2_ films (although it should be noted that the VO_2_ wires are approximately 50 nm thicker than the earlier films due to fabrication differences). These same fabrication differences may also be the cause of the slightly lower MD reported for sample IV below 0.7 THz (see Fig. 5(c)).

#### Electrically switching on/off behaviors

**Figure 6:**
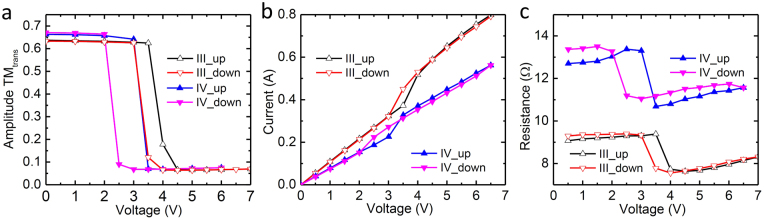
Electrical characterizations of sample III and sample IV. Measured amplitude transmission at 1 THz (**a**), R-V (**b**) and I-V (**c**) curves during the voltage going up and down cycles for sample III (black and red hollow triangle curves) and IV (blue and pink triangle curves), respectively.

To have a close observation of the electrical switching on/off behavior of the metallic bowtie-VO_2_ hybrid device, the amplitude transmission for TM modes at 1 THz with voltage ramp up and ramp down for sample III and sample IV are plotted in Fig. 6(a). We observe the hysteresis of the switching processes, with an approximately 0.5 V/1.0 V difference between the 50-percent point of the switching on and switching off curves for sample III (black and red hollow triangles in Fig. 6(a)) and sample IV (blue and pink triangles in Fig. 6(a)), respectively. This electrical hysteresis behavior is quite similar to those observed in thermally activated VO_2_ films which may be due to the transition-triggering nucleation sites appearing at different voltages depending on whether the voltage is ramping up or down^39^. Additionally, the transition voltages for both switching on and switching off processes shift to lower voltages upon changing the configuration of VO_2_ from film to wire (Fig. 6(a)). This is probably due to the change in the quantity of the surface defects, typically oxygen vacancies in metal-oxide material surface, which act as nucleation sites for the phase transition and from the modification of the in-plane tensile stress of the VO_2_ wires compared to the bare film^40^. The increase in the surface-to-volume ratio in VO_2_ wire leads to a dramatic increase in the number of surface defects as well as nucleation density for the same volume of VO_2_ film. Hence, the required density of transition-triggering nucleation sites can be achieved at a lower voltage, resulting in a lower phase transition voltage for the VO_2_ wires.

The decrease in the transition voltage from sample III to sample IV is also shown in the I-V curves (Fig. 6(b)). For the voltage ramp up, an abrupt jump in the current for sample III (black triangle curve on Fig. 6(b)) is observed when the voltage increases from 3.5 to 4 V, whereas the current jump for sample IV occurs 0.5 V lower, between 3 and 3.5 V (blue triangle curve in Fig. 6(b)). For voltage ramp down, the sharp drop in the current for sample III (red triangle curve in Fig. 6(b)) is accompanied by decreasing the voltage from 3.5 to 3 V, whereas sample IV has the same drop approximately 1 V lower, from 2.5 to 2 V (pink triangle curve in Fig. 6(b)). The larger transition voltage change in the ramp off process leads to the broadening of the hysteresis in sample IV. This broadening may also be attributed to the higher density of surface defects in the VO_2_ wire than in the VO_2_ film, leading to a higher density of nucleation sites as well as metallic domains persistence after the IMT. As a result, more correlated electrons will be trapped and localized by the defects and dislocations among the domains; consequently the relaxations of them require a larger decrease in the voltage to trigger the sharp decrease in current during the MIT process. The gradients of the I-V curves in Fig. 6(b) show that the resistance of sample IV has increased compared to sample III, which is shown in Fig. 6(c). The higher resistance of sample IV (blue and pink triangles in Fig. 6(c)) is mainly due to the lower coverage of VO_2_ wires on the sapphire substrate, increasing the effective resistivity of the device. Thus if we keep on reducing the dimension of VO_2_, even higher resistances can be achieved. As a result, our hybrid metallic bowtie-VO_2_ devices are feasible in maintaining a stable and safe operation status for real applications.

#### Time responses of the device

As a multifunctional device, besides the modulation depth and modulation bandwidth, another valuable element to be considered is the modulation speed. The amplitude variation of the peak to peak signal of the THz time domain pulse is recorded when the voltage was applied directly from 0 to 6 V for ramp on process and then from 6 to 0 V for the ramp off process. Two distinguishable periods for each process are observed: one is the turn on/off delay time indicating the time delay between voltage on/off and THz amplitude change, the other is the time used for changing the peak to peak signal from maximum to minimum and vice versa (the switch on/off time). The time responses of sample IV are shown in the first row of Table 2 with the corresponding periods of sample I listed below for comparison. Sample I was chosen as the representative for comparison since its time responses behave similarly for the switching on process as compared with sample II and III, but slightly faster (~2 s) for the switching off process. The significant improvement lies in the switching on time of sample IV which is more than 4 times faster than that of sample I. This improvement is attributed to the reduced dimension of VO_2_ wire leading to the faster arrival of the critical charge density under the applied electric field for which the monoclinic to rutile phase transition occurs^41^. In addition, a dramatic increase in the turn off delay time is observed in sample IV. That’s because firstly during the switching off process, the strongly correlated electrons dissipate through randomly diffusion^42^ rather than the directional electrical driven; Secondly, the diffusion is restricted along the wire rather than widespread in every direction in the VO_2_ film; Thirdly, large amounts of electrons trapped in the VO_2_ wire due to its large density of defects greatly prolongs the electron relaxation process before triggering the metal to insulator state transition. This inspires a possible way to reduce the turn off delay time, that is, to keep on reducing the dimension of VO_2_ wire. For example, the VO_2_ wires can be replaced by smaller VO_2_ squares or rectangles, and located exactly underneath the gaps between the square bowties. By this way, the number of metallic domains as well as localized electrons can be greatly reduced and be easily dissipated resulting in less diffusion and relaxation time. Application of the electric field would then rapidly induce the IMT, shorting the contacts and developing a wire grid structure to block the hitherto unhindered TM THz polarized wave. Upon switching off the electric field, the small number of domains would quickly return to the insulator state. To push a step further, one can imagine if only single domain is included in VO_2_, the time delay induced by interactions among the domains would be negligible, thus the transition would be in an instantaneous way. The other element influencing the switching on/off time is the thickness of VO_2_ with thinner film leading to higher transition speed. The switching time in microseconds scale has been reported in 100 nm thick and 20 *μ*m length VO_2_ square^43^. Hence, it is possible to control the modulation speed of the THz signal by tuning the configuration and dimension of the VO_2_ film, and without significant compromise of the modulation depth since the square bowtie metallic array with small gap is less dependent on the VO_2_ conductivity (Fig. 3(c)). Despite the relatively low modulation speeds, similar devices to this could find use as a wave modulator or absorber in a situation where only occasional switching is needed.

**Table 2:** Time responses of the bowtie antanna-VO_2_ hybrid devices. Turn on/off delay time is the time delay between the voltage and terahertz amplitude responses.

Sample type	Turn on delay time (s)	Switch on (s)	Turn off delay time (s)	Switch off (s)
IV	3	3	18	10
I	1	13	8	9

## Conclusions

In conclusion, a multifunctional THz modulation device was proposed and experimentally demonstrated. It consists of VO_2_ active layers on the bottom and bowtie metallic arrays on the top, such that the electrically driven VO_2_ phase transition can be realized by applying an external voltage between adjacent metal wires extended to a large area array. A broadband MD response from 0.3 to 2.5 THz has been achieved, benefiting from the THz frequency independent properties of VO_2_. Different increases in the MD for different metallic array designs indicate that the gap size between the bowties is crucial in controlling this enhancement effect, with smaller gap leading to higher MD and *Δ*_MD_. Moreover, the MD of the square bowtie with a 1 *μ*m gap exhibits the least dependence on the VO_2_ conductivity, thereby making it possible to keep a high modulation efficiency even after reducing the thickness of VO_2_ film. By changing the configuration of VO_2_ from film to wire, the switch on time improves by 4–5 times, implying that higher modulation speed can be achieved in smaller and thinner VO_2_ pieces. The prolonged switch off time in the wire configuration is probably due to the random diffusion of electrons being highly confined in the narrow wires leading to a much slower relaxation process before triggering the metal to insulator phase transition. The different time responses in different configurations of VO_2_ imply that the high tunability of modulation speed can be further explored for specific applications.
